# A live-cell super-resolution technique demonstrated by imaging germinosomes in wild-type bacterial spores

**DOI:** 10.1038/s41598-020-62377-1

**Published:** 2020-03-24

**Authors:** R. M. P. Breedijk, J. Wen, V. Krishnaswami, T. Bernas, E. M. M. Manders, P. Setlow, N. O. E. Vischer, S. Brul

**Affiliations:** 1 Van Leeuwenhoek Centre for Advanced Microscopy, Molecular Cytology, Swammerdam Institute for Life Sciences, University of Amsterdam, Amsterdam, Science Park 904, 1098 XH The Netherlands; 2 Molecular Biology and Microbial Food Safety, Swammerdam Institute for Life Sciences, University of Amsterdam, Amsterdam, Science Park 904, 1098 XH The Netherlands; 3 Department of Anatomy and Neurobiology, Virginia Commonwealth University, PO Box 980709, 1101 East Marshall Street, Richmond, VA 23298 United States of America; 4 Confocal.nl B.V., Science Park 106, Amsterdam, 1098 XG The Netherlands; 5 Department of Molecular Biology and Biophysics, UConn Health, 263 Farmington Avenue, Farmington, CT 06030-3305 United States of America

**Keywords:** Bacterial development, Bacterial structural biology, Imaging and sensing, Confocal microscopy, Super-resolution microscopy

## Abstract

Time-lapse fluorescence imaging of live cells at super-resolution remains a challenge, especially when the photon budget is limited. Current super-resolution techniques require either the use of special exogenous probes, high illumination doses or multiple image acquisitions with post-processing or combinations of the aforementioned. Here, we describe a new approach by combining annular illumination with rescan confocal microscopy. This optics-only technique generates images in a single scan, thereby avoiding any potential risks of reconstruction related artifacts. The lateral resolution is comparable to that of linear structured illumination microscopy and the axial resolution is similar to that of a standard confocal microscope. As a case study, we present super-resolution time-lapse imaging of wild-type *Bacillus subtilis* spores, which contain low numbers of germination receptor proteins in a focus (a germinosome) surrounded by an autofluorescent coat layer. Here, we give the first evidence for the existence of germinosomes in wild-type spores, show their spatio-temporal dynamics upon germinant addition and visualize spores coming to life.

## Introduction

For many years, fluorescence has been widely used for microscopic imaging of biological specimens. The quest for generating images with improved resolution and contrast has resulted in the development of super-resolution imaging techniques^1–7^. These techniques produce images with a lateral resolution that is beyond the diffraction limit^1,4,6^. A study on the applicability of such techniques for practical bio-imaging purposes has been reported recently^7^. Each of these techniques have their own merits and limitations. Techniques like single molecule localization microscopy (SMLM) and structured illumination microscopy (SIM) rely on acquisition of multiple images that are mathematically post-processed to reconstruct a super-resolution image^8–10^. To ensure a successful reconstruction, it is essential to acquire images that cater to the needs of the processing algorithm. For example, in SMLM, resolution of the reconstructed image is primarily governed by the number of detected photons from each emitter^11^. Therefore, multiple image acquisitions are required and time resolution is compromised. In addition, fluorescence signals are usually weak in live-cell imaging scenarios, where over-expression of fluorophores is avoided to prevent biological artifacts^12–15^. Compared to SMLM, SIM requires far less images for its reconstruction and provides a two-fold increase in resolution over conventional microscopy. However for SIM it is imperative to acquire high quality images with an optical transfer function that is perfectly aligned with the processing algorithm. A successful image reconstruction requires the user to understand the raw data set and appropriately configure the settings for the applied reconstruction algorithm^7^. Despite this, the SIM reconstruction can be susceptible to image artifacts. For example, motion related artifacts can be generated from dynamic events within the specimen that occurs during the image acquisition sequence^5,16,17^. The applicability of SIM is limited particularly when the fast dynamic movements are larger than half the obtainable image resolution. On the other hand a technique like stimulated emission depletion microscopy (STED) uses an optics-only approach to generate super-resolution images in a single scan^18^. However, its universal applicability to live-cell imaging scenarios with physiological expression still remains a question due to the requirement of high illumination doses for its operation^19^. Furthermore, photo-dynamic and thermal effects can interfere in the imaging process, consequently compromising image veracity^20^. Therefore, a more friendly live-cell imaging super-resolution technique is long desired. Here, we propose an optics-only based super-resolution imaging approach that does not require mathematical post-processing or high illumination doses for its operation. An artifact-free super-resolution image can be generated in a single scan, by using an annular aperture in the excitation path of a rescan confocal microscope. This technique scores high on its ease of use and can be applied to any common confocal imaging scenarios with standard probes and sample preparation routines.

Confocal microscopy has long been a reliable workhorse for high-contrast fluorescence imaging. It uses a pinhole in its detection path to improve image contrast. A pinhole with size smaller than 1 Airy unit (AU) can be used for generating images with improved lateral resolution, at the cost of reduction in the signal-to-noise ratio (SNR). Theoretically, a 1.4 fold improved resolution over widefield microscopy can be recorded with an infinitely small pinhole. In practical situations, the SNR may be insufficient to register the improved resolution. To solve this problem, the dependency of lateral resolution and the pinhole size needs to be decoupled. For this purpose, rescan confocal microscopy (RCM) can be used. It incorporates the principle of image scanning microscopy (ISM)^21–26^. Here, the 1.4 fold improved lateral resolution can be achieved with a pinhole of any size^25^. The principle of RCM therefore allows for super-concentration of light resulting in improved SNR^27,28^. In addition, the axial resolution is also improved by a factor of 1.15 ± 0.08^28^.

With RCM, the resolution is still limited by the dimensions of the excitation point spread function (PSF). Here, we explore the use of an annulus to engineer the excitation PSF to smaller lateral dimensions. Past studies have established that an annular aperture generates an excitation PSF with a narrower central lobe but with more pronounced lateral side lobes and axial lobes^29–31^. In theory, a 1.75 fold improved lateral resolution over widefield microscopy should be possible^29^. However, a standard confocal microscope would need an infinitely small pinhole to realize the improved resolution. For the case of absorptive based imaging of gold nanoparticles, researchers have reported a lateral resolution with full width at half maximum (FWHM) of 122 nm (405 nm excitation)^32^. For the case of fluorescence imaging, similar results have remained a challenge^32^. Here, we combine annular illumination with RCM to realize a 1.75 fold improvement in lateral resolution over widefield microscopy, with fluorescence imaging. Compared to RCM without annulus this factor is 1.25. This achieved lateral resolution is comparable to that of linear SIM. On the other hand, the axial resolution is worse when annular illumination is used with standard confocal microscopy. This is compensated for by the rescan effect which enhances the axial resolution by a factor of 1.15 ± 0.08. Therefore, by combining RCM with annular illumination, the lateral resolution is improved, without significant loss in axial resolution when compared to standard confocal microscopy.

The proposed technique is broadly applicable to several live-cell imaging scenarios. We present imaging of germinosomes in bacterial spores as a case study. Germinosomes are clusters of germinant receptor (GR) proteins which trigger the awakening of spores of the model organism *Bacillus subtilis*^33,34^. There are only low numbers of GRs in such spores and their visualization is hampered by the highly autofluorescent coat layer. In order to image germinosome dynamics, three main challenges have to be addressed. Firstly, it is essential to capture as much signal as possible from the weakly fluorescent signals from GRs to boost the SNR. Secondly, it is essential to visualize the GRs in greater detail despite the presence of autofluorescence from the coat layer by improving image contrast. Thirdly, it is essential to select an imaging technique that is suitable to capture any potential fast spatio-temporal dynamics of the GRs without any artifacts. Thus far, researchers have circumvented the first two challenges by mutating spores (*cotE gerE* mutants), or by chemically removing the outer coat layer to reduce autofluorescence^35^. However, altering the coat alters spore physiology, slowing germination and decreasing spore heat resistance^35^. Notably, while the organization and morphology of proteins in the coat of wild-type *Bacillus* spores were visualized with ellipsoid localization microscopy, no such reports exist for germinosomes in ‘wild-type’ *Bacillus* spores^36,37^. Recent efforts to visualize germinosomes in *Bacillus subtilis cotE gerE* mutants using SIM were not successful^33^. Performing SIM imaging with high SNR and time resolution (faster than the movements of GRs) that is required for a successful mathematical reconstruction proved challenging. Therefore, an alternate super-resolution microscopic technique to image wild-type bacterial spores is desirable. Our imaging approach uses super-concentration of light and a pinhole to readily provide images with improved SNR and contrast. This addresses the first two imaging challenges. The third challenge is addressed by adopting a point scanning approach. Here, spatial sampling is performed within a short span of time, and information recorded by each pixel is only influenced by a small area surrounding it. Consequently, motion blur is minimized and the movement of spots is captured within successive frames with a certain update frequency^38^. In addition, no mathematical post-processing is required and therefore our technique is immune to any image reconstruction artifacts. By addressing these challenges, we perform time-lapse, super-resolution imaging of wild-type bacterial spore germination using our new imaging approach.

## Results

### Super-resolution imaging configuration

**Figure 1:**
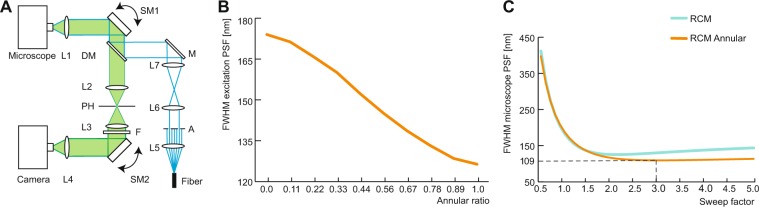
Optical setup and design considerations. (**A**) Schematic of an RCM setup with annular illumination. Excitation light from the fiber is collimated by lens L5 and directed to an annular obstruction target (A) which is placed at the focal point of lens L6. The combination of L6 and lens L7 is a magnifying telescope. The light is directed onto a scanning mirror pair (SM1) through a mirror (M) and the dichroic mirror (DM). The focal point of the scan lens L1 is placed at the telecentric plane in between the 2 scanning mirrors of SM1. The focal plane of L1 coincides with the image plane of the microscope. Fluorescence emission from the microscope is descanned by SM1 and directed to a pinhole (PH) via DM and lens L2. Lens L3 collects the emission light from the PH and directs it to a second pair of scanning mirrors (SM2) through an emission filter (F). The rescanned light is collected by rescan lens L4, which forms an image at its focal distance which is captured by a camera. (**B**) Plot of lateral FWHM of the main lobe of the excitation PSF relative to the annulus size. (**C**) Plot of lateral FWHM of the microscope PSF vs. the sweep factor.

The optical illumination and detection paths of a standard confocal microscope are adapted to perform super-resolution imaging (Fig. 1A). Illumination is performed with a 488 nm laser source with an annular obstruction target placed in the optical excitation path. Detection is performed with the RCM principle, using a re-scanning unit and a camera in the emission path. With RCM, it is essential to determine a sweep factor (M), the ratio between the angular amplitudes of the rescanning and scanning mirrors^25^. The optimum corresponds to $M=1+{({W}_{em}/{W}_{ex})}^{2}$, where ‘*W*_*em*_’ is the FWHM of the emission PSF and ‘*W*_*ex*_’ is the FWHM of the excitation PSF. In case of annular illumination, the size of the annulus has a direct influence on the resulting ‘*W*_*ex*_’. We use the term ‘annular ratio’, describing the size of the annulus as the ratio between the outer diameter of the annular obstruction target to the diameter of the objective entrance pupil. Higher annular ratios yield smaller ‘*W*_*ex*_’ (Fig. 1B). We used a 100x/1.49NA objective with an annular ratio of 0.8, the upper limit of our optical setup. When the annular ratio was greater than 0.8, a certain fraction of the scanned excitation beam was lost at the entrance pupil of the objective, thereby limiting the total scanning area. The scanning field area is maximum when the X and Y scanning mirrors are placed at exactly the telecentric point of the scan lens. However, in our setup the telecentric point was located right in between the X and Y scanning mirrors to avoid the use of relay optics that reduces the detection efficiency of the emission signal. An alternate solution is to use a static beam for illumination while using a stage scanning approach for point-wise imaging. However, in this case the imaging speed is reduced and therefore we discarded this approach.

We estimated the value of *W*_*ex*_ =  134 nm (488 nm excitation) based on the simulations of our optical setup, while *W*_*em*_ =  187 nm (525 nm emission) was calculated from Abbe’s diffraction limit equation. As a result, an optimal *M* = 3.0 was calculated and with these settings, the calculated lateral resolution was found to be 109 nm (Fig. 1C). It is noteworthy that annular illumination requires a 1 Airy unit (AU) pinhole to mask the more pronounced side lobes of excitation PSF. This ensures that the emission signal originating from the side-lobes excitation is effectively blocked from detection. Also, the difference between excitation and emission PSFs contribute to reduction of the effect of side lobes of the former. Due to a smaller central lobe of the excitation PSF, the physical size of the 1 AU pinhole is smaller than 1 AU pinhole used for conventional confocal imaging. The lateral resolution is maintained for pinhole sizes ≤1*AU* in accordance with the principle of RCM.

### Microscope characterization

**Figure 2:**
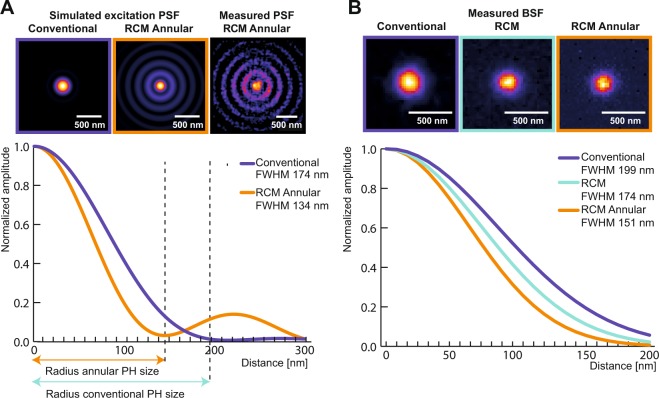
Excitation PSF and microscope BSF in the lateral dimension. (**A**) Simulated images of excitation PSF from conventional and annular illumination are shown. For annular illumination, the simulated spatial profile matched with the measured profile that was recorded using the bead scanning technique. Plot from simulation data shows the difference in excitation profiles of conventional and annular illumination. (**B**) Comparison of measured BSFs of confocal, RCM and RCM Annular with a 1 AU pinhole each, using circularly polarized excitation in all cases.

**Figure 3:**
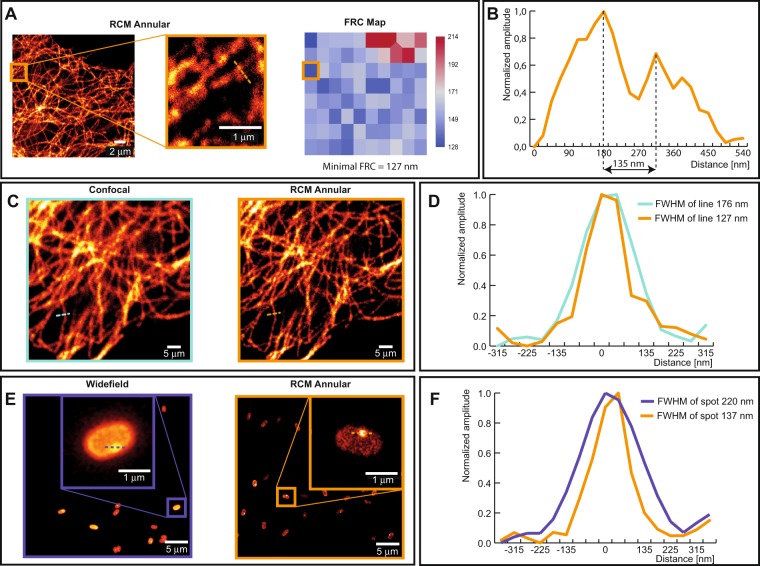
Images of tubulin and a *Bacillus subtilis* spore using conventional approaches and RCM with annular illumination. (**A**) RCM Annular image of a tubulin along with a corresponding map of Fourier ring correlations (FRC) obtained by using the ImageJ NanoJ-SQUIRREL plugin. (**B**) Line profile plot indicating feature seperation of two tubulin filaments by a distance of 135 nm. (**C**) Comparison of a confocal image (left) and an RCM Annular image (right) of tubulin stained with Alexa 488 in HeLa cells. (**D**) Measured FWHM from the spatial intensity profile of a tubulin. (**E**) Comparison of a widefield Fluorescence image of an RCC PS832_GerKB-sGFP dormant spore (left) and RCM Annular microscopy (right). (**F**) GerKB-sGFP in a wild-type spores were visualized as a spot with a FWHM 137 nm by RCM Annular microscopy.

We visualized the spatial profile of the excitation PSF using a bead scanning approach^39^. The recorded lateral profile of the excitation PSF corroborated data generated from numerical simulations (Fig. 2A). The resolution of a confocal microscope is measured by detecting the microscope PSF, which is a combination of both excitation and emission PSF. For this purpose, we imaged 100 nm green fluorescent beads and 60 nm silver beads. Here, we recorded the bead spread function (BSF) which is a standard measure of the actual PSF with a known bias, that depends on the physical size of the bead^40^. Analytical expressions were then used to calculate the BSF based on the theoretical PSF^40^. Another factor that influences the imaging resolution is the polarization of the excitation beam^41–43^. With circular polarization, we measured an isotropic lateral FWHM of 153 ± 11.7 nm (Fig. 2B) from the 100 nm beads. With linear polarization, non-isotropic lateral resolutions down to lateral FWHMs of 174 ± 9.2 nm and 136 ± 19.2 nm were recorded in horizontal and vertical directions, respectively (see Fig. S1 in Supplementary information). We selected circular polarization for our imaging experiments owing to its superior photoselection properties for fluorescence excitation. The measured BSFs were found to be in line with the theoretical estimates (see Table S1 in Supplementary information). This was also the case with reflective imaging of 60 nm silver beads where a lateral FWHM of 126 nm was recorded (see Fig. S2 in Supplementary information). Similar measurements and calculations were performed for determining the axial FWHM (see Fig. S3 in Supplementary information and Table S2 in Supplementary information). Here, we focus on 2D imaging of bacterial spores, which are relatively thin in our case. In addition, to compare our simulation results with measured data, we imaged tubulin stained with Alexa 488 in HeLa cells. The minimum Fourier ring correlation (FRC) resolution was measured to 127 nm (Fig. 3A). Spatial features of tubulin down to 135 nm were resolved (Fig. 3B). In addition, we compared the images of the same field of view, using both confocal and RCM with annular illumination (Fig. 3C). The lateral FWHM of the tubulin was measured to 176 nm in the case of confocal microscopy, whereas a lateral FWHM of 126 nm was obtained from images recorded using our technique (Fig. 3D).

### Time-lapse, super-resolution imaging of wild-type bacterial spores

Our imaging method offers improved contrast and resolution. We applied this method to visualize the dynamics of the GR GerK B-subunit (GerKB) fused to sGFP in a wild-type *Bacillus subtilis* background; GRs recognize nutrient germinants and initiate germination. The GerK and GerB GRs are localized in the spore inner membrane (IM) in germinosomes and cooperate to sense asparagine, glucose, fructose and potassium (AGFK) to initiate germination. Compared to wild-type sprores, no significant deviation in the time to germination was found in our GerKB-sGFP mutant in AGFK induced germination at single spore level (data not shown). A previous study also indicated that GerK is the rate-limiting protein in this process^44^. The GerKB-sGFP fusion proteins appeared as a spot of a lateral FWHM of 137 nm, which was clearly visualized with our imaging method, despite the coat autofluorescence (Fig. 3E,F).

**Figure 4:**
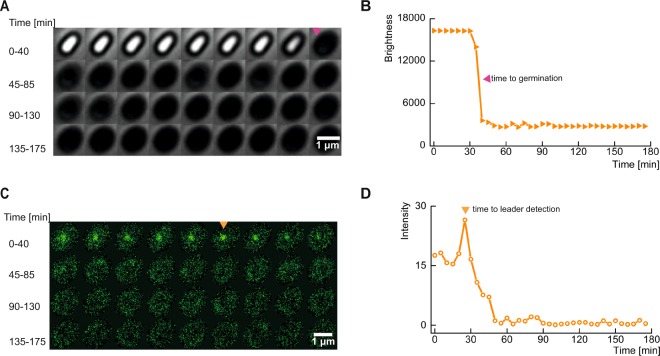
History of a single rich culture condition (RCC) spore on an AGFK-MOPS agarose pad. (**A**) Montage of time-lapse frames in phase contrast. The magenta arrow indicates germination. (**B**) The brightness in phase contrast drops upon spore germination. (**C**) Fluorescence montage; the most prominent spot (‘leader’) is indicated by the orange arrow. (**D**) Fluorescence of a GerKB-sGFP spot increases prior to germination and then drops rapidly.

We recorded phase contrast and fluorescence time lapse images to study the dynamics of the GerKB proteins during germination (Fig. 4A,C). Germination is accompanied by a change in the spore core refractive index due to the loss of the large core depot of CaDPA (a 1:1 chelate of Ca^2+^ and dipicolinic acid [DPA]) and water uptake, leading to a significant drop in the phase contrast image brightness (Fig. 4B). Here, CaDPA is released via the SpoVA CaDPA channel, which is stimulated by signals from germinant activated GRs. In the fluorescence channel, a series of 8 raw fluorescence images were acquired every 5 min at equal time intervals with a scanning time of 2 sec per image. Here, most of the GerKB-sGFP proteins were visualized as spots (germinosomes!) in the fluorescence images with FWHMs between 91–263 nm (see Fig. S4 in Supplementary information). A slow scanning rate of 7.11 mm/sec was used in combination with a low illumination instantaneous peak power of 15.5 kW/cm^2^ for imaging to minimize photobleaching. Several nongerminanting spores with GerKB proteins’ spots in the fluorescence channel persisted without a significant drop in intensity throughout the imaging period, which testifies to negligible impact of photobleaching (see Fig. S5 in Supplementary information). Raw images were recorded with low SNR and therefore the 8 images were combined by image averaging to generate a single image that represents the activity in the fluorescence channel with a 5 min time resolution (see Movie S1 in Supplementary information). We also noted that the fluorescence intensity of the GerKB proteins’ spots fluctuated significantly within the 5 min intervals. In addition, we visualized 8 specific spores with two spots in the resulting images, but we considered the spot with greater intensity, relative to the cell background for our analysis (see Movie S2 in Supplementary information). We speculate that these intensity fluctuations in the raw images are due to reversible GerKB interactions with other germinosome proteins. We plan to address this possibility in our future work. When we analyzed the averaged images, most GerKB proteins’ spot(s) persisted for several min beyond the completion of spore germination. Notably, before the germination event, we observed a significant increase in the fluorescence intensity of the spot(s), relative to the cell background (Fig. 4D). The most prominent spot (hereafter referred to as ‘leader spot’) has been detected around the spore phase transition (Fig. 4C,D). This phenomenon suggests a change in GerKB-sGFP structure or its environment upon germinant binding. A subsequent drop in spot intensity after spore phase transition was observed. We confirmed that no new protein synthesis was observed in this period by Western Blot analysis (see Fig. S6 in Supplementary information). We speculate that germinant activated GRs in the germinosome need to oligomerize before activating the SpoVA channel for DPA secretion and core water uptake, again a topic for future research^45^.

**Figure 5:**
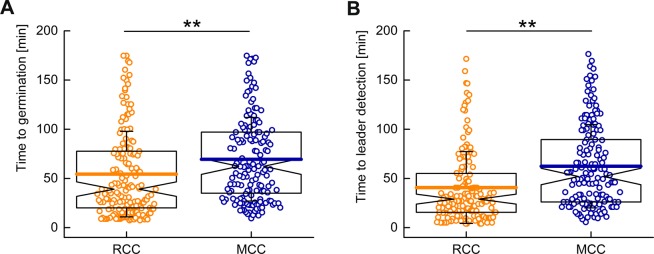
Comparison of germination dynamics between RCC and MCC spores. (**A**) The majority of the RCC spores (54.4 ± 43.5 min) germinated faster than MCC spores (69.4 ± 42.5 min). (**B**) Peak fluorescence intensities were observed earlier for RCC spores (40.7 ± 36.5 min) in comparison to MCC spores (62.4 ± 42.3 min). Here t = 0 represents the beginning of the imaging experiment. In the box plot, the whiskers represent standard deviation, the horizontal line in the box represents mean value, and notches on the black box indicate the median value. Significant differences were measured by the Mann-Whitney rank sum test (**p < 0.01).

To study the quantitative influence of GR proteins on the time to germination, we prepared spores in two different sporulation media; a rich culture condition (RCC) and a minimal culture condition (MCC). We observed more RCC spores germinating when compared to MCC spores within the observation time period of 180 min (see Fig. S7 in Supplementary information). In addition, the germination events for RCC spores occurred 15 min faster than for MCC spores (Fig. 5A). This result was consistent with inferences from previous studies at a population level. GRs are responsible for the “awakening” of the spores and hence significantly influence germination rates^46^. Therefore, it was no surprise that RCC spores exhibited higher fluorescence intensity, relative to the cell background, than MCC spores (see Fig. S8a,b in Supplementary information). Similar inferences could be drawn for the detection of leader spots during the germination process (Fig. 5B).

**Figure 6:**
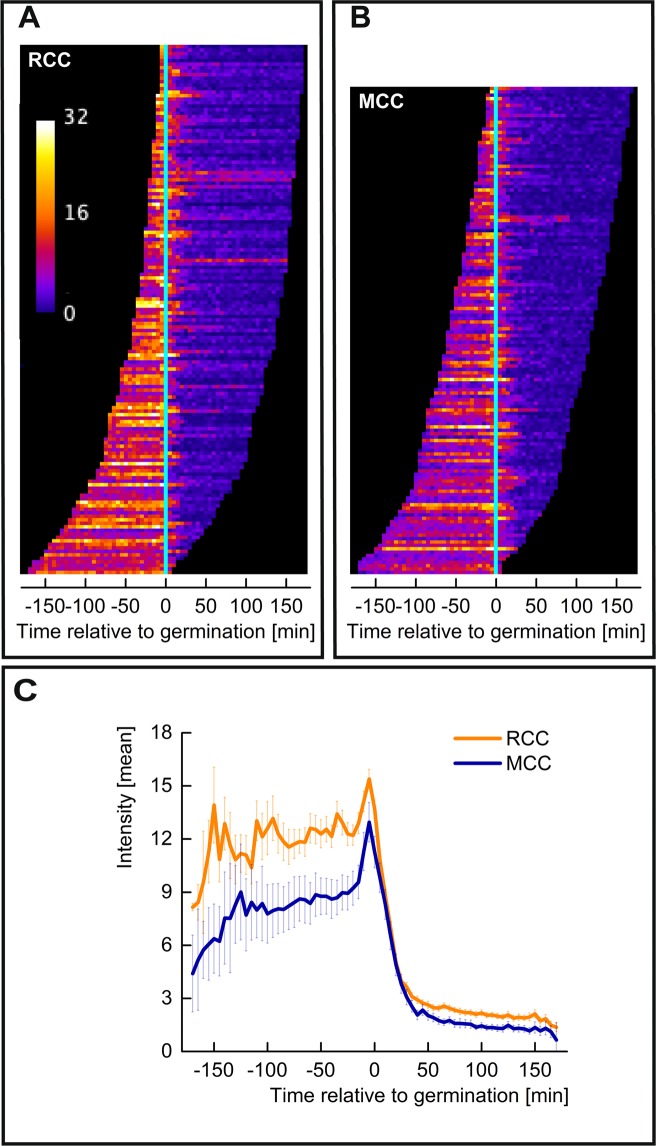
Dynamics of germinosomes during germination. (**A**,**B**) Map of Leaders displays the germinosome dynamics of RCC and MCC spore populations, respectively. For each spore, sorted from early to late germination, the leader history is displayed as one row of pixels. Here t = 0 represents the time of germination. (**C**) Collective profiles of RCC and MCC are obtained by vertical average-projection of the respective map. Error bars correspond to 95% confidence.

Interestingly, of all the spores that germinated and in which a GerKB-sGFP spot could be detected, both the RCC and MCC spores reached maximum GerKB-sGFP fluorescence around the phase transition. In both populations, the intensity of GerKB-sGFP spots relative to the cell background dropped after the phase transition as shown in the map of leaders (Fig. 6A–C). While previous studies reported a correlation between the amounts of GRs and the time to germination at a population level, we found no clear correlation at a single spore level^44^. This suggests a more complex mechanism involving additional molecular components as the basis of germination heterogeneity^45^.

To analyze this heterogeneity further, the germination process was studied within two time intervals. The first interval is between the beginning of the sample preparation time and the time at which the leader spot is detected. The second interval is the time between leader spot appearance and the germination event. For most spores, the leader spot was detected before the germination event. In addition, leader spots were detected earlier in the majority of the RCC spores when compared with MCC spores (Fig. 5B). However, the time interval between the leader spot and the germination event was similar in both the populations. This suggests that, in the case of MCC spores, activated complexes of GerKB-sGFP are formed slower.

## Discussion

Unique time-resolved images of germinosomes in *Bacillus subtilis* wild-type spores allowed us to interrogate the initial molecular details of spore germination in greater detail. Previous studies have indicated that the majority (almost 70%) of lipids in the inner membrane (IM) of *Bacillus subtilis* are immobile and are accompanied by a small fraction of mobile lipids^47^. After germination, both the IM mobile fraction and lipid diffusion coefficient increases^47^. We observed fluctuating fluorescence signals from the GerKB-sGFP spots within 5 min time intervals over a specific area, even in the absence of any nutrient supplement. This suggests that the IM mobility is low, while the mobile lipids may be concentrated in specific areas where the GerKBs are located. Such areas are likely segmented or surrounded by immobile lipids, thereby restricting the area in which GerKB molecules can be present. In addition, we speculate that the nutrients induce oligomerization of GRs resulting in stronger fluorescence of GerKB spots, before CaDPA release via the SpoVA CaDPA channel. It is possible that nutrient germinant addition triggers increased IM fluidity around the germinosome, thereby allowing the GerKB proteins to move around.

Previous studies have indicated that the spore initially commits to germination, which is then followed by a rapid release of CaDPA during the germination process^48^. The commitment to the germination process is marked by slow leakage of CaDPA with increased permeability of the spore’s IM. Commitment ensures that germination is unstoppable, despite the removal of nutrients or inhibition of further germinant binding to GRs^48^. The slow leakage of CaDPA probably occurs via the SpoVA channel, indicating the partial activation of SpoVA channel by GRs prior to the commitment^48^. Such processes can now be explored with this new imaging technology.

Our super-resolution imaging technique does not require any specific sample preparation routines, exogenous fluorophores, high power lasers or computational power. It is architecturally less complex compared to other super-resolution microscopic techniques and easy to use. Standard preparation protocols and imaging expertise in using a standard confocal microscope would suffice. In terms of imaging performance, this technology can be directly compared to linear SIM with respect to resolution (see Table S3 in Supplementary information). However, the speed limitations of this technique can be equated to any advanced point-scanning technique such as confocal or STED microscopy. Today, with the advent of fast resonant scanners, video-rate imaging can be realized with point-scanning systems^49^. Therefore, in principle, artifact-free super-resolution images at video-rate can be generated by using this optics-only approach, without the requirement for any mathematical reconstruction. The use of a pinhole is a plus to achieve improved contrast and hence resolution optically, thereby making it useful for imaging thick specimens. While the use of an annular aperture for illumination leads to an elongated PSF in the axial direction, there is little or no loss of axial resolution when compared to standard confocal imaging, due to the rescan effect. The axial resolution degrades only when the elongation of the PSF is greater than 1.23 times to that of a standard PSF obtained with standard confocal excitation. Therefore, the extended depth of focus with annular imaging can be used to image thick tissues with improved resolution^31^. This technique can also be combined with two-photon or multi-photon excitation approaches, that are more commonly used for deep-tissue imaging. Another consequence of using annular illumination is the occurrence of more pronounced side lobes intensity of the excitation PSF. From an imaging point of view, these undesired side lobes contribute to photodamage and also restrict the size of the pinhole used in the detection path. However, photodamage can be alleviated by ensuring illumination dose control, using fluorescent probes with high photostability, and by stabilizing the microenvironment of the specimen^50^. In addition, by changing the size of the annulus, imaging trade-offs between lateral-resolution and SNR can be addressed. Therefore, the size of the annulus used for imaging should be carefully selected, depending on the requirements of the imaging experiment.

Apart from the choice of annulus size, the choice of polarization state of the excitation beam is important. A circularly polarized beam with annular illumination can be used to obtain a narrow, symmetric distribution of excitation light in the objective focus^32,43^. One may note that a narrower PSF can be obtained when a radially polarized beam is used instead. But, for a circularly polarized beam a transverse polarization is obtained in the center of the focus, while a longitudinal polarization is obtained in the case of a radially polarized beam^43^. The opposite situation is encountered in the side lobes surrounding the central maximum^43^. Since one can expect emission dipoles of fluorescent labels being colinear with their excitation dipoles (in fixed biological specimen), circular polarization provides higher excitation efficiency along the central lobe^51^. The converse is true in case of side lobes around the central maximum. This effect diminishes the advantage of the radially polarized beam in a scenario where the fluorescence signal of bound molecules is detected. Moreover, using this modality, one may not neglect differences in refractive indices (between medium and components of the cells) in the imaged specimen. The presence of such interfaces may significantly distort the focus of a radially polarized beam, while the focusing of circularly polarized light is less sensitive to the differences in the effective numerical aperture^43^. Consequently, for practical fluorescence-based bio-imaging, a circularly polarized excitation beam is likely to perform better than its radially polarized counterpart.

## Methods

### Custom microscope

We built an RCM microscope for imaging (Fig. 1A). The excitation beam from a 488 nm laser (Coherent OBIS 488LS20) is directed to a polariser (CVI Melles Griot, 03FPG003) and a half wave plate (Thorlabs, AHWP05M-600) to generate a linearly polarized laser beam. An additional quarter wave plate (Thorlabs, AQWP05M-600) is used to generate circular polarization when necessary. To generate annular illumination, polarized light is guided through an annular aperture obstruction target (Thorlabs, R1CA800). A telescopic system is used with two lenses, L6 (f = 30 mm) and L7 (f = 80 mm), to project the annular aperture onto the back focal plane of the objective. The beam is then guided onto a multiband dichroic mirror (Chroma ZT405/488/561/640rpc) and then to a scanning mirror unit (Thorlabs GVS002). The beam is then directed on to the scan lens that projects the light into the image plane of the microscope body. Two different microscope bodies were used for imaging, the Nikon TE2000 (for bead measurements) and the Nikon Ti-E (timelapse measurements). The Nikon 100x Apo Tirf SR 1.49 was used for all fluorescence imaging measurements, while a 100x phase contrast objective (Plan Apo 100x/1.45 phase 3) was used for phase contrast measurements. The emission light from the objective is descanned and focused with lens L2 (f = 80 mm) onto a 30 *μ*m pinhole (Thorlabs P30D). Light from the pinhole is collimated by lens L3 (f = 60 mm) and guided to a rescanning mirror unit (Thorlabs GVS002) via an emission filter (Chroma ZET405/488/561/640). The rescanner directs the light to a camera through rescanning lens L4 (f = 75 mm). An sCMOS camera (Hamamatsu Flash 4.0) was used in conjunction with the Nikon TE2000 microscope for imaging tubulin samples, while two EMCCD cameras (Andor888 Ultra and Andor iXon897) were used for time lapse fluorescence and phase contrast imaging respectively. A Nidaq card (National Instruments Co. PCI-6733) and LabVIEW 2012 are used for electronic triggering and control of scanning units. NIS Elements (Nikon Instruments) was used for image data acquisition and microscope control. For the purpose of comparison, imaging was also performed with a conventional confocal microscope (A1 Nikon Instruments).

### Simulation model

The excitation PSF of the microscope objective (NA = 1.49), corresponding to an excitation wavelength of 488 nm, is calculated using vector diffraction theory, based on Richards and Wolf integrals and later developed by Youngworth and Brown for polarized light^52,53^. Plane wave illumination, circularly polarized light and aplanatic imaging is assumed in the calculations, as established in a previous study^43^. The objective aperture is either clear (full) or its center is blocked with an annular amplitude mask. For an annular ratio of 0.8, the width of the annulus corresponds to 20% of the aperture radius. The emission PSF (525 nm) is calculated for a clear aperture and linear polarization, averaged over all possible orientations to simulate non-polarized fluorescence emission. Images of PSF cross sections (XY and XZ) are rendered with 1.45 nm pixel size. Effects of the sweep factor (1x–4x) and the pinhole size (0.5 AU–2.0 AU) on the RCM and confocal imaging were examined using a simulation procedure established earlier^25^. Here, the object plane was discretized with a 425 × 425 grid of 1.45 nm mesh size. A single point source was placed in the center and then convolved with the product of excitation and emission PSFs. The resulting object image was scanned with the pinhole (diameter determined by full excitation PSF) in the X and Y dimensions with a step size of 1.45 nm. The RCM image is then generated by projecting the pinhole onto the image plane (discretized at 1.45 nm) with steps of 1.45 nm–5.80 nm (depending on the sweep). The confocal image was generated by projecting an integral of the pinhole with 1.45 nm step size (sweep 1.0x). To match the magnification, images were scaled to a pixel size of 1.39 nm. FWHMs of the system PSFs of the point source were measured along a linear profile through final images of the point source.

### Bead scanning

To visualize the lateral profile of the illumination pattern, we used a bead scanning process, as established previously^39^. We used a 100 nm fluorescent bead on the XY-stage of the microscope. While the scanning mirrors are parked at their central position, the laser beam is guided to the bead which is moved in X and Y directions, with a step size of 75 nm. At every position an image is recorded and analyzed using the rapidStorm localization algorithm. The recorded image at each position is localized to remove the effect of the emission PSF, thereby providing a super-resolution image of the annular illumination pattern (Fig. 2A).

### Image acquisition and processing

Time lapse fluorescence and phase contrast imaging of spores was performed for 180 min. While a single phase contrast image was acquired once every 5 min, a series of 8 fluorescence images were acquired within the same interval. The scanning time for each fluorescence image was 2 sec and an illumination power of 12.5 *μ*W (measured at the objective) was used for imaging. This resulted in the application of a instantaneous peak power density equivalent to 15.5 kW/cm^2^.We used a mechanical objective switching mechanism to use objectives with and without phase rings for recording phase contrast and fluorescence images respectively. Therefore, significant amounts of drifts were recorded in the raw images. The spatial drifts were corrected for the phase contrast images using a template matching plugin that is available in Fiji/ImageJ. For the fluorescence images, images are first processed using Fiji/ImageJ to identify pixels affected by detection of cosmic rays. Such pixel values are replaced by the median intensity value of the image. Then, an average of 8 fluorescence images was computed to represent fluorescence activity within the 5 min interval. For correcting the spatial drifts, template registration was first performed on a bandpass filtered image to ensure that noise does not interfere with the registration process. Then, the resulting translational shifts that were recorded were reapplied to the raw images. Next, the field of view from both the phase contrast and fluorescence channels had to be aligned. Phase contrast and fluorescence images were recorded using two optical paths and ports each equipped with a different camera. Therefore, the pixel sizes of the images were different. To match the pixel sizes, phase contrast images were scaled proportionally to match the raw data in the fluorescence channel. In addition, the fluorescence images were rotated by an angle of −2.8° to compensate for the angular difference between the camera mounts. A bandpass filter was applied to the first fluorescence image and its template was matched with the first phase contrast image. The recorded translational shifts were then applied to all phase contrast images, to ensure aligned fields of view in both channels. The common region of interest that was well registered in both channels at all time points was selected and cropped to generate a hyperstack comprising 36 time frames at 5 min intervals for further analysis. The preparation times of several min, needed to mount the specimen on the microscope, were recorded as metadata and considered during analysis. It has to be noted, that these image pre-processing steps can be avoided or minimized depending on the optical imaging configuration set up used for phase contrast imaging.

### Image analysis

The analysis protocol was implemented with ImageJ and the ObjectJ plugin. For each population, about 8 movies were linked to an ObjectJ project file (‘.ojj’ extension), which is located in the same folder as the movies. It integrates embedded macros for analysis, navigation and creation of plots and contains derived results and the landmarks for all linked movies. Throughout the analysis, the image files are displayed as virtual stacks and remain unchanged, while filters and other modifications only have temporary effects. Identification of spores is done with particle analysis on a median-filtered copy of the first phase contrast time frame. Spore centers are marked in frame #1, close neighbors were removed where necessary. For subsequent analysis, two montage stacks (phase contrast and fluorescence) are created and stored. This concept allows the observation of the entire history of one spore at a time as a dual montage showing 4 × 9 = 36 snapshots per channel, including landmarks for germination and fluorescent foci (Fig. 4A,C). Spores that show a significant bright-to-dark transient at their center are assumed to germinate where the intensity drops below 50% of the entire range. Germination is visualized as a stack of plots showing brightness vs. time, for each spore in phase contrast (Fig. 4B) and fluorescence (Fig. 4D). The fluorescence montage was temporarily smoothed (sigma = 67 nm) and its background subtracted. The respective background image was created by applying an appropriate median filter to suppress the peaks. Next, the most prominent peak (‘leader’) of all 36 snapshots was detected. A circular region around its center was used to evaluate fluorescence versus time at the leader position throughout all time frames. A radius of 225 nm was chosen to tolerate the slight spot movements. The integrated peak fluorescence is assumed to be proportional to the mean intensity of the circle. Fast user navigation allows browsing through all spores in synchrony with the related results and plots. For either population, the collective germinosome dynamics relative to the time point of germination is displayed as a ‘Map of Leaders’ (Fig. 6A,B). Here, non-germinating spores and those with very weak leaders are excluded.

### Sample preparation

#### Beads

FluoSpheres carboxylate-modified microspheres, 100 nm, yellow-green fluorescent (505/515), 5% solids, azide free (ThermoFisher Scientific, F8803), mounted in mowiol embedding medium, and 60 nm silver dispersion nanoparticles, 0.02 mg/mL in aqueous buffer with sodium citrate as a stabilizer (Sigma-Aldrich, 730815) were prepared in accordance with an established protocol^54^.

#### Hela-tubulin sample

To stain tubulin, HeLa cells were grown for 24 hr on cleaned #1.5 coverslips in Dulbecco’s Modified Eagle Medium (DMEM). The cells then were fixed with 10% 2-(N-morpholino) ethanesulfonic acid (MeS) buffer (100 mM MeS, pH 6.9, 1 mM EGTA and 1 mM MgCl_2_) and 90% methanol for 5 min on ice. After blocking with 5% Bovine Serum Albumin (BSA) for 1 hr, cells were incubated with rabbit anti-tubulin polyclonal antibodies (Abcam) for 1 hr. Subsequently, all cells were incubated with goat anti-rabbit Alexa antibodies (Alexa 488, Invitrogen) for 30 min. All fixation and staining steps were done at room temperature.

#### *Bacillus subtilis* spores

Strain PS832_GerKB-sGFP was obtained by fusing the sGFP coding sequence to the C-terminal coding end of the *gerKB* gene by a single crossover in the background of *Bacillus subtilis* PS832, a prototrophic 168 laboratory strain. The native *gerKB* is most likely not transcribed in this construct, only the gene encoding the GerKB fusion protein. Spores of *Bacillus subtilis* strains were prepared either in MOPS minimal medium or 2xSG rich medium using established methods. After 2 days (2xSG) or 3 days (MOPS), spores were harvested and purified using established methods^55^. Spores (OD600 = 60) were stored at −80 °C prior to experiments. Spores (OD600 = 30) were incubated for 1 hr at 70 °C for heat-activation, then cooled on ice for 15 min. Spores were immobilized on a 1.5% agarose matrix, supplemented with MOPS medium and the spore germinant AGFK mixture (10 mM L-asparagine, 28 mM glucose, 28 mM fructose and 50 mM potassium chloride), in a gene frame slide using established methods^56^.

#### Western blot

Spore germination was induced by AGFK in MOPS medium as mentioned above. Samples of dormant, germinating 15 min, germinating 30 min, and germinating 60 min spores were collected to prepare spore lysates using an established procedure^57^. Proteins from equal aliquots of the same amounts of spores were separated by Tricine-SDS-PAGE, and probed with rabbit polyclonal anti-GFP antibody (Abcam), using established procedures^58^.

### Accession codes

SporeTrackerB analysis software is available online at https://sils.fnwi.uva.nl/bcb/objectj/examples/sporetrackerb/MD/SporeTrackerB.html

## Supplementary information

Supplementary Information.

Supplementary Information2.

Supplementary Information3.
