# Supplementary material for: A live-cell super-resolution technique demonstrated by imaging germinosomes in wild-type bacterial spores
Source: Sci Rep. 2020 Mar 24;10:5312. doi: 10.1038/s41598-020-62377-1 (PMC7093444; doi:10.1038/s41598-020-62377-1)
Supplement: Supplementary file 1 — Supplementary Information. [file 41598_2020_62377_MOESM1_ESM.pdf]

# A live-cell super-resolution technique demonstrated by imaging germinosomes in wild-type bacterial spores

**R.M.P.Breedijk[1,+], J.Wen[2,+], V.Krishnaswami[1,+], T.Bernas[3], E.M.M.Manders[1,4], P.Setlow[5], N.O.E.Vischer[2], and S.Brul[2,*]**

[1]Van Leeuwenhoek Centre for Advanced Microscopy, Molecular Cytology, Swammerdam Institute for Life Sciences, University of Amsterdam, Amsterdam, Science Park 904, 1098 XH,The Netherlands.
[2]Molecular Biology and Microbial Food Safety, Swammerdam Institute for Life Sciences, University of Amsterdam, Amsterdam, Science Park 904, 1098 XH, The Netherlands.
[3]Department of Anatomy and Neurobiology, Virginia Commonwealth University, PO Box 980709, 1101 East Marshall Street, Richmond, VA, 23298, United States of America.
[4]Confocal.nl B.V. Science Park 106, Amsterdam, 1098 XG, The Netherlands.
[5]Department of Molecular Biology and Biophysics, UConn Health, 263 Farmington Avenue, Farmington, CT 06030-3305, United States of America.
[*]s.brul@uva.nl
[+]these authors contributed equally to this work

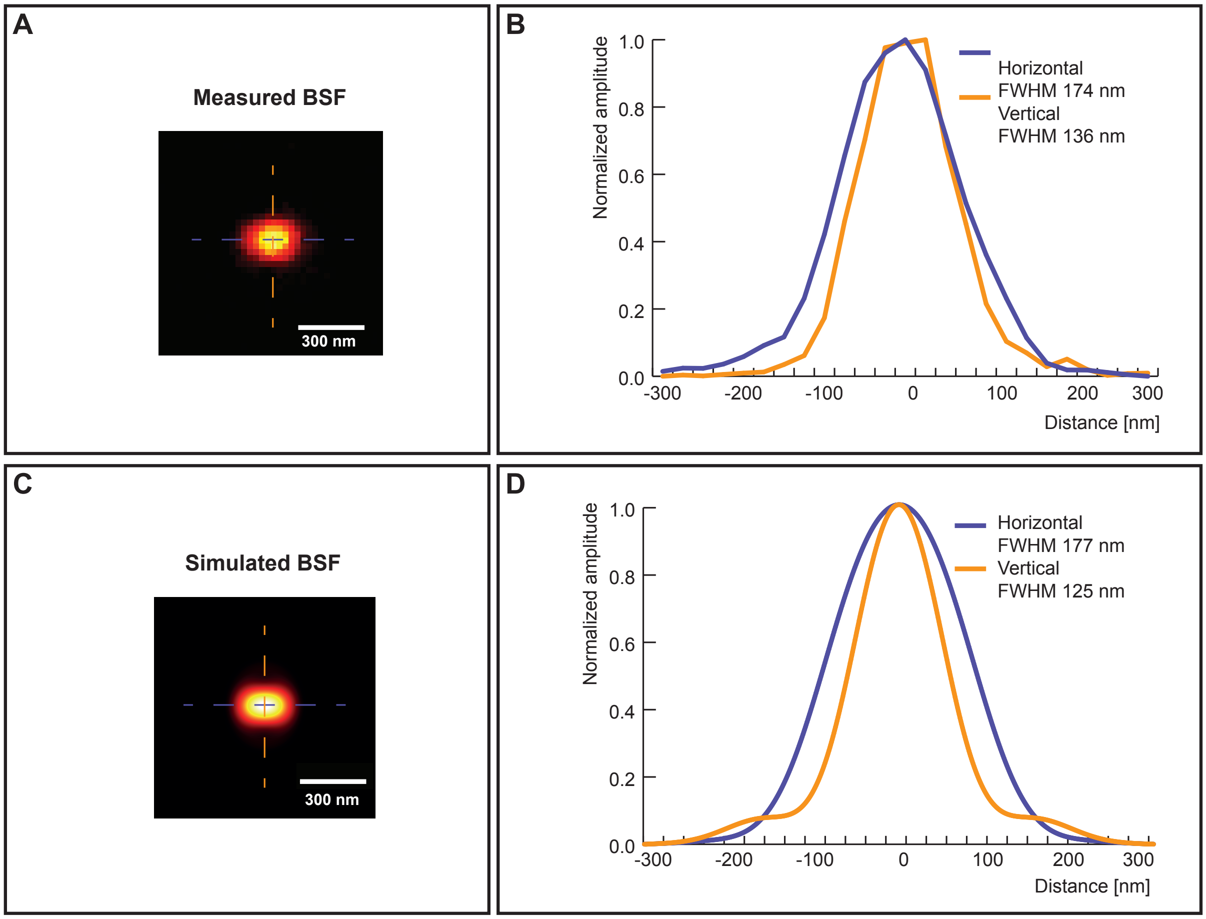


**Figure S1. Lateral BSF with linearly polarized excitation. (A)** Recorded BSF when imaging was performed using annular illumination with linearly polarized excitation. **(B)** Measured spatial intensity profile of the BSF along the horizontal and vertical axes. Notably non-isotropic resolutions were measured in horizontal and vertical directions. The measured BSF is in agreement with the simulated data as shown in **(C)** and **(D)**.

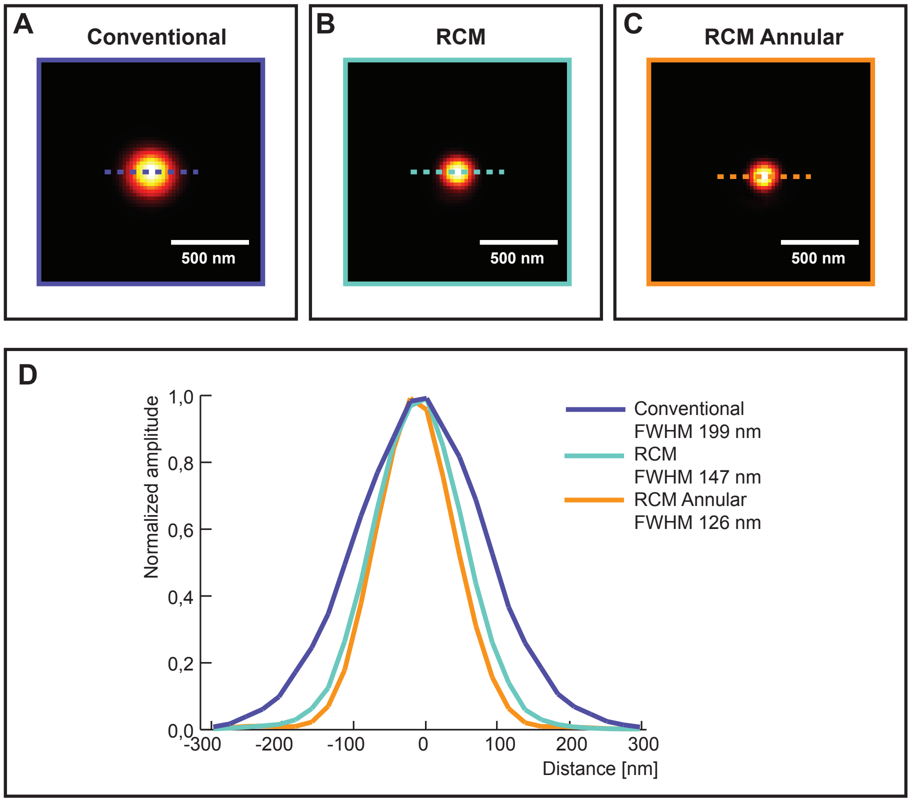


**Figure S2. Lateral BSF measurements using 60 nm silver beads.** **(A)** Reflective imaging of 60 nm silver beads, performed using conventional confocal microscopy, **(B)** RCM without an annulus and **(C)** RCM with an annulus. **(D)** Line profiles of these three cases.A FWHM of 126 nm was recorded using RCM with annular illumination which is about 1.2 fold better than RCM imaging and 1.6 fold better than conventional confocal imaging.

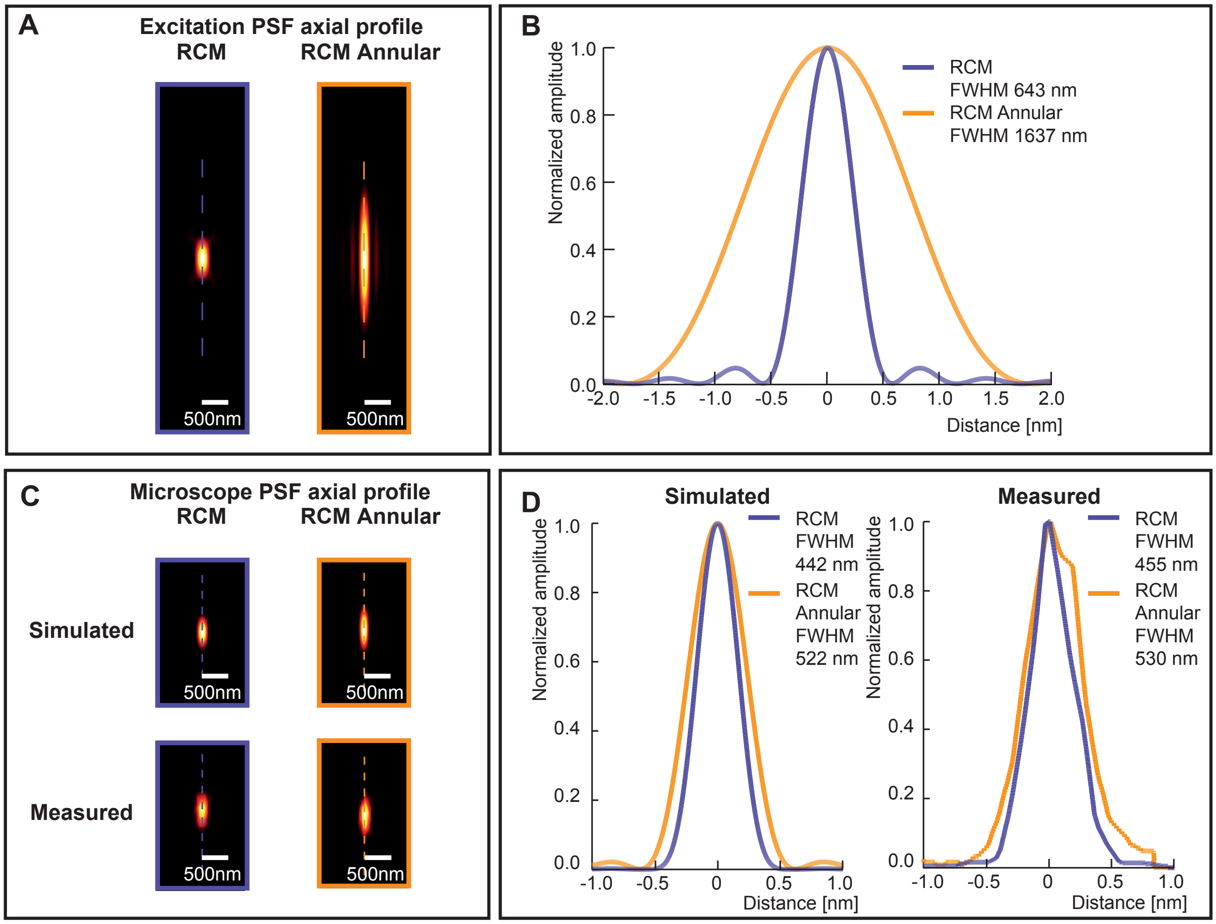


**Figure S3.** **Simulation of axial BSF.** **(A)** From simulation we compared the axial profile of the excitation PSF without and with an annulus. **(B)** Using an annular ratio of 0.8 resulted in a 2.5x larger PSF in the axial direction, compared to conventional imaging. **(C)** and **(D)** When a 1 AU pinhole was used for detection, the axial resolution was degraded by a factor of 1.4. The measured data matched the simulations.

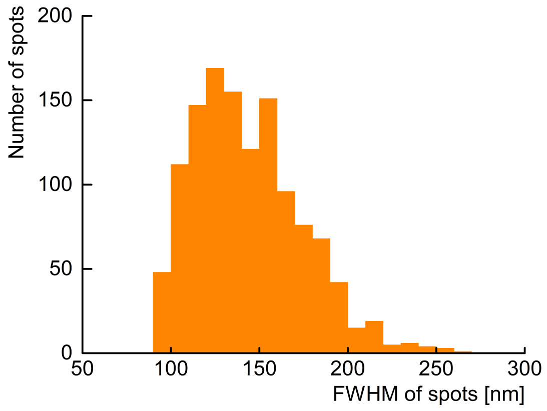


**Figure S4. Spot size measurements.** Histogram showing lateral FWHM of GerKB-sGFP spots (143 ± 30 nm) of PS832_GerKB-sGFP spores exposed to AGFK measured from raw fluorescence images. These raw images were smoothed using a mean based kernel (radius = 45 nm) using ImageJ, to minimize the impact of noise on the FWHM measurements. Prominent spots were identified from all the time-lapse datasets and fitted to a gaussian profile to calculate radial FWHM. The histogram includes only measurements with a gaussfit $r^2 > 0.8$.

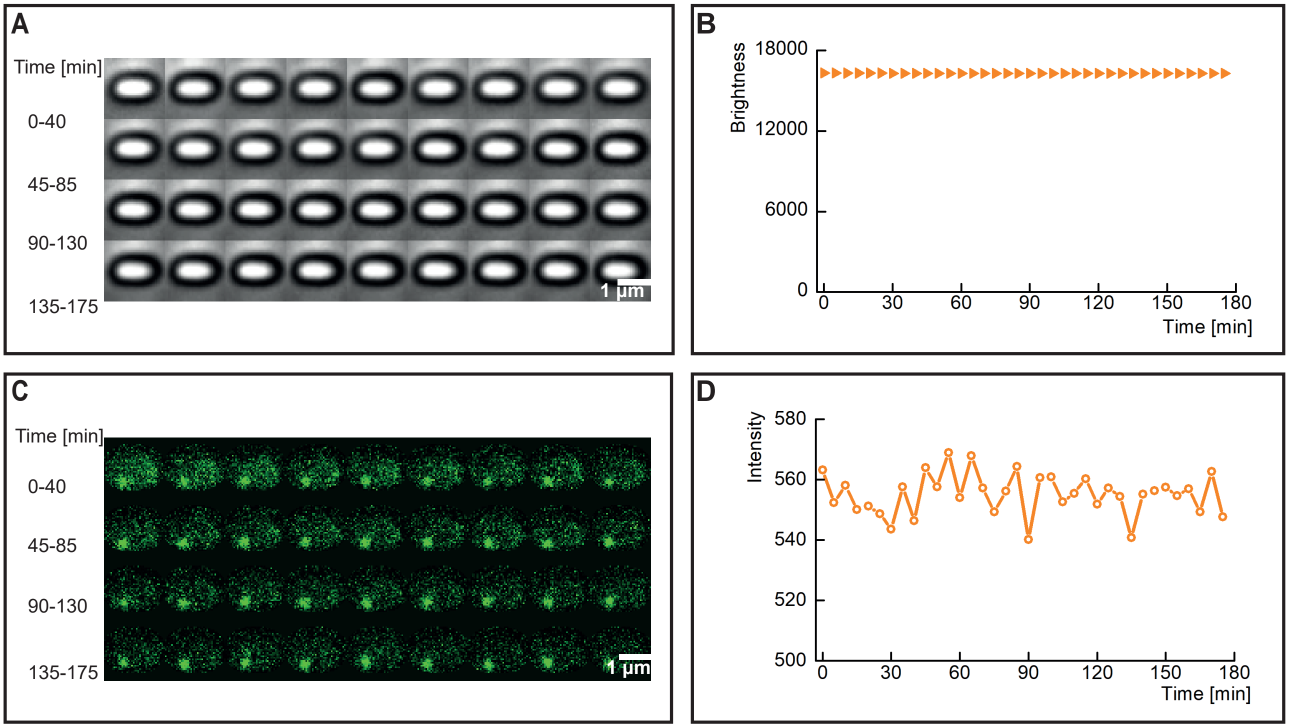


**Figure S5. History of a single RCC dormant spore on an AGFK-MOPS agarose pad. (A)** Montage of time-lapse frames in phase contrast. **(B)** The plot profile of brightness in the phase-contrast matrix. **(C)** Fluorescence montage. **(D)** The plot profile of GerKB-sGFP spot intensity in the fluorescence phase-contrast matrix. The fluorescence intensity fluctuations does not drop significantly over the imaging duration of 180 min, suggesting negligible photobleaching.

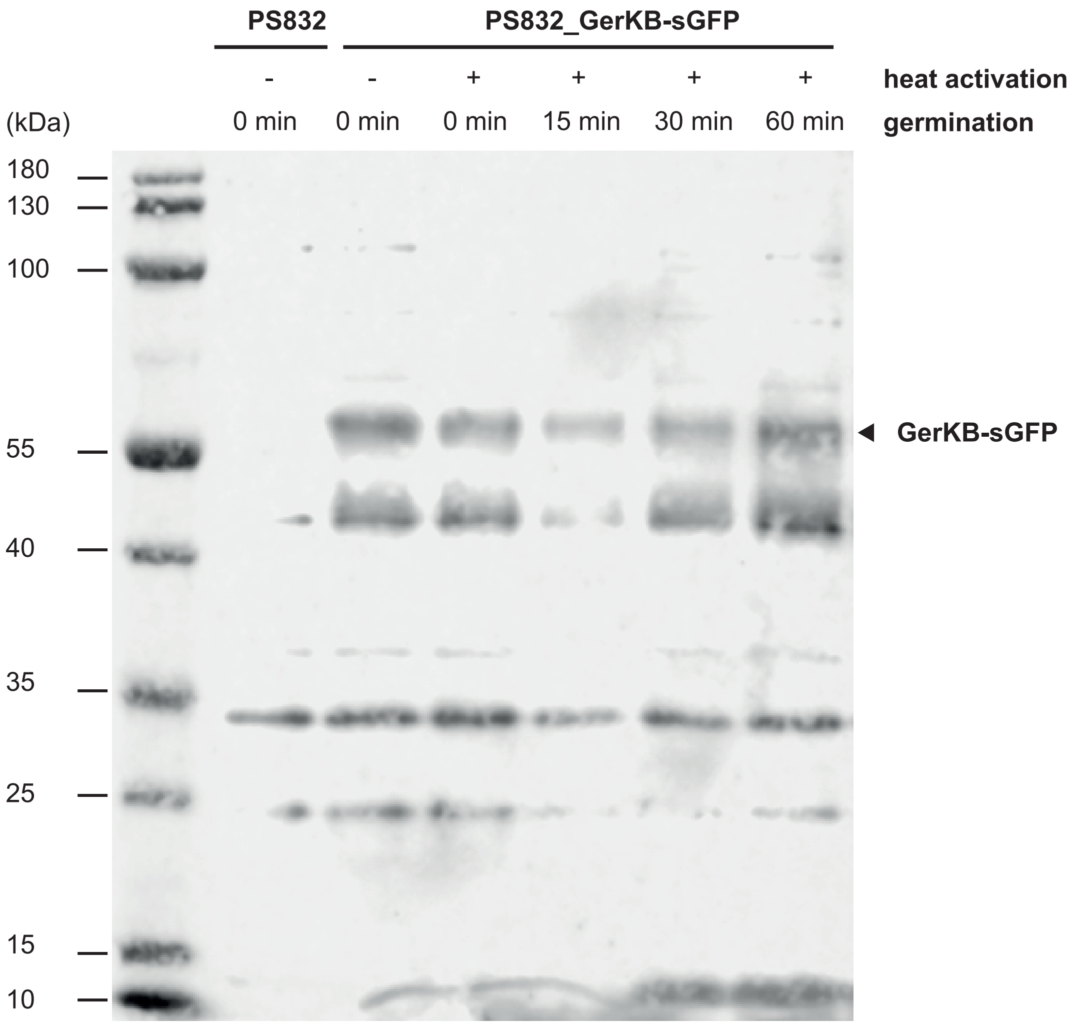


**Figure S6. Western blot of GerKB-sGFP in dormant and germinating MCC PS832 and PS832_GerKB-sGFP spores.** PS832_GerKB-sGFP spore germination was induced by AGFK after heat activation at 70°C for 1 h. Germinating PS832_GerKB-sGFP spores were collected after 0, 15, 30 and 60 min of germination. Proteins from equal aliquots of the same amounts of dormant and germinating MCC PS832 and PS832_GerKB-sGFP spores were separated by a Tricine-SDS-PAGE gel, and probed with polyclonal rabbit anti-GFP antibodies (Abcam). The band with the predicted molecular weight for GerKB-sGFP is indicated with an arrow and was clearly only present in the PS832_GerKB-sGFP spores. No anti-GerKB-sGFP cross reactive material was detected in wild type PS832 spores. The band below the GerKB-sGFP band could be a degradation product of GerKB-sGFP. Three unspecific bands at lower molecular weight regions cross-reacted with the antiserum in both PS832 and PS832_GerKB-sGFP spore extracts.

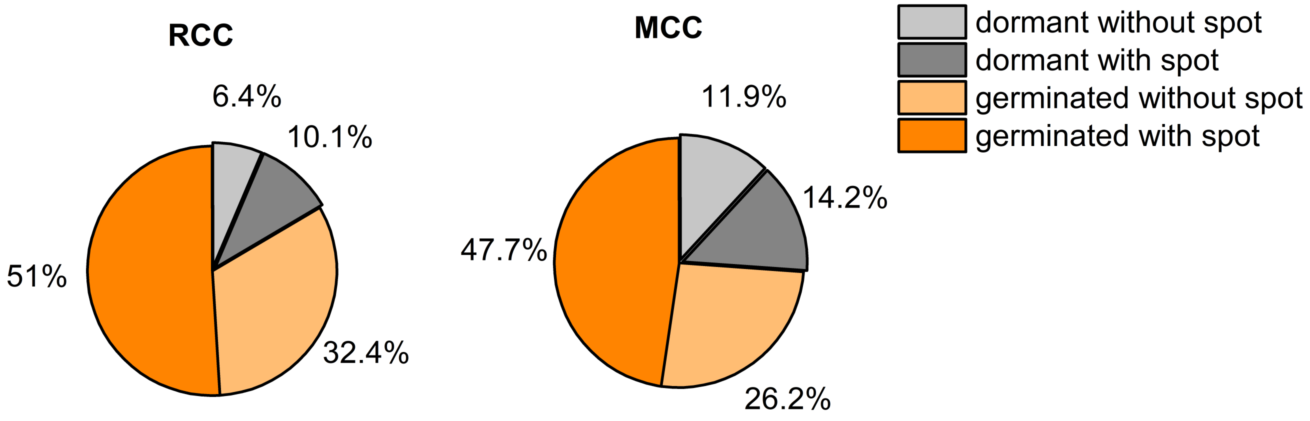


**Figure S7.** **Statistical measurements of germination events and spot detection.** A greater number of PS832_GerKB-sGFP RCC spores (left) germinated when compared to MCC spores (right) within the observation period of 180 min. In total, 296 RCC spores and 302 MCC spores were tracked by ImageJ with SporeTrackerB. Spores that developed a peak above a chosen threshold during the observation time were qualified as "with spots". Spores were sorted into four subpopulations based on dormant/germinated and with/without GerKB-sGFP. Spores, which completed germination and had spots, were plotted in Fig. 5, Fig. 6, and Fig. S8.

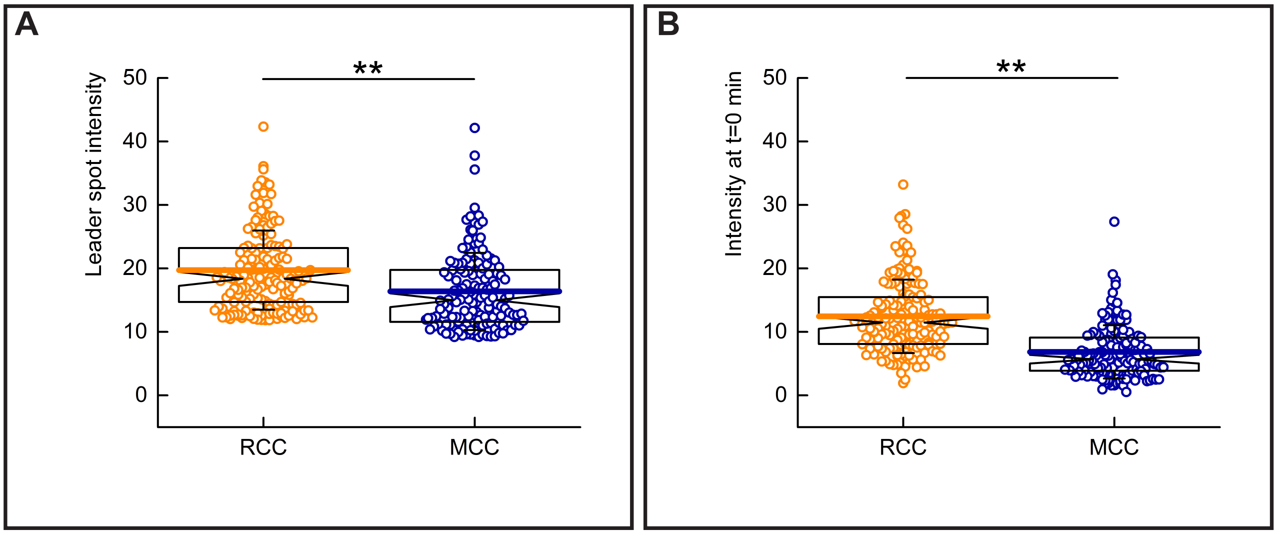


**Figure S8.** **Comparison of spot intensities between RCC and MCC spores.** **(A)** The majority of germinating RCC PS832_GerKB-sGFP ($19.7 \pm 6.2$) spores exhibited higher leader intensities than MCC spores ($16.4 \pm 6.1$). **(B)** A similar trend was observed at the beginning of the imaging experiment (t=0 min). In the box plot, the whiskers represent standard deviation, the horizontal line in the box represents mean value, and notches on the box indicate the median value. Significant differences were measured by the Mann-Whitney rank sum test ($**p < 0.01$).

| Lateral resolution (FWHM) | Theory (point source) nm | Calculated$^{+}$ (100nm beads) nm | Measured$^{*}$ (100nm beads) nm | Calculated$^{+}$ (60nm beads) nm | Measured (60nm beads) nm |
|---|---|---|---|---|---|
| Confocal (1AU) | 167 | 195 | 196 | 196 | 199 |
| RCM | 135 | 168 | 171 | 148 | 147 |
| RCM annular | 109 | 148 | 153 | 128 | 126 |

**Table S1.** Comparison of lateral resolution based on FWHM measurements from 100 nm fluorescent and 60 nm silver beads. $^{+}$Refers to the calculated value of FWHM that takes into account the size of the bead, based on Theer et al., 2014. $^{*}$Measurements performed using circular polarization, for linear polarization refer Fig. S1.

| Axial resolution (FWHM) | Theory (point source) nm | Measured $^{*}$ nm |
|---|---|---|
| Confocal (1AU) | 526 | 517 |
| RCM | 449 | 455 |
| RCM annular | 528 | 530 |

**Table S2.** Comparison of axial resolution based on FWHM measurements from beads. $^{*}$Measurements performed using circular polarization.

| Criterion | Localization microscopy$^{+}$ | STED$^{+}$ | Linear SIM$^{+}$ | RCM-annular | RCM |
|---|---|---|---|---|---|
| Resolution | *** | *** | ** | ** | * |
| Sample preparation | ** | ** | *** | *** | *** |
| System complexity | *** | * | ** | *** | *** |
| Ease of imaging | ** | * | *** | *** | *** |
| Imaging fast dynamic movements | * | *** | ** | *** | *** |
| Acquisitions required | * | *** | ** | *** | *** |
| Image reconstruction issues | ** | *** (!) | * | *** (!) | *** (!) |

**Table S3.** Comparison of different super-resolution imaging approaches. $^{+}$Is largely adapted from Wegel et al., 2016. (!)Adopts an optics only approach and there is no requirement for image reconstruction.
